# Epidemiology of respiratory infections among adults in Qatar (2012-2017)

**DOI:** 10.1371/journal.pone.0218097

**Published:** 2019-06-13

**Authors:** Hamad Eid Al-Romaihi, Maria K. Smatti, Nandakumar Ganesan, Shazia Nadeem, Elmoubasher Farag, Peter V. Coyle, Joanne Daghfal Nader, Hebah A. Al-Khatib, Emad B. Elmagboul, Said Al Dhahry, Salih A. Al-Marri, Asmaa A. Al Thani, Abdullatif Al Khal, Muna A. Al Maslamani, Hadi M. Yassine

**Affiliations:** 1 Ministry of Public Health, Doha, Qatar; 2 Biomedical Research Center, Qatar University, Doha, Qatar; 3 Hamad Medical Corporation, Doha, Qatar; 4 Hamad Bin Khalifa University, Doha, Qatar; 5 College of Health Sciences, QU Health, Qatar University, Doha, Qatar; Vetsuisse Faculty of the University of Bern, SWITZERLAND

## Abstract

**Background:**

Limited data is available about the etiology of influenza like illnesses (ILIs) in Qatar.

**Objectives:**

This study aimed at providing preliminary estimates of influenza and other respiratory infections circulating among adults in Qatar.

**Methods:**

We retrospectively collected data of about 44,000 patients who visited Hamad General Hospital clinics, sentinel sites, and all primary healthcare centers in Qatar between 2012 and 2017. All samples were tested for influenza viruses, whereas about 38,000 samples were tested for influenza and a panel of respiratory viruses using Fast Track Diagnostics (FTD) RT-PCR kit.

**Results:**

Among all ILIs cases, 20,278 (46.5%) tested positive for at least one respiratory pathogen. Influenza virus was predominating (22.6%), followed by human rhinoviruses (HRVs) (9.5%), and human coronaviruses (HCoVs) (5%). A detection rate of 2–3% was recorded for mycoplasma pneumonia, adenoviruses, human parainfluenza viruses (HPIVs), respiratory syncytial virus (RSV), and human metapneumovirus (HMPV). ILIs cases were reported throughout the year, however, influenza, RSV, and HMPV exhibited strong seasonal peaks in the winter, while HRVs circulated more during fall and spring. Elderly (>50 years) had the lowest rates of influenza A (13.9%) and B (4.2%), while presenting the highest rates of RSV (3.4%) and HMPV (3.3%). While males had higher rates of HRVs (11.9%), enteroviruses (1.1%) and MERS CoV (0.2%), females had higher proportions of influenza (26.3%), HPIVs (3.2%) and RSV (3.6%) infections.

**Conclusion:**

This report provides a comprehensive insight about the epidemiology of ILIs among adults in the Qatar, as a representative of Gulf States. These results would help in improvement and optimization of diagnostic procedures, as well as control and prevention of the respiratory infections.

## Introduction

Acute respiratory infections (ARIs) lead to significant rate of mortality and morbidity worldwide. According to the World Health Organization (WHO), approximately 290,000 to 650,000 deaths annually are caused by influenza virus infection alone [1]. Several other respiratory pathogens including human rhinoviruses (HRVs), respiratory syncytial virus (RSV), human parainfluenza viruses (HPIVs), human metapneumovirus (HMPV), adenoviruses, enteroviruses, and human coronaviruses (HCoVs) are associated with the development of ARIs [2]. Nevertheless, due to the similarity in the clinical presentation of these infections, the particular underlying cause is usually difficult to be identified unless with laboratory testing; and generally, ARI patients are subjected to presumptive treatment [3].

Although the epidemiology and seasonality of influenza and other respiratory viruses are well characterized in many regions of the world, limited data is available from the Middle East and North Africa (MENA) region [4–7]. Importantly, accurate data on the incidence and epidemiological distribution of respiratory viruses is significant to the general public health, and have important implication on disease prevention and control. Thus, national surveillance systems for influenza and ILIs have been implemented in several countries [8–10].

In the state of Qatar, there is a diverse and rapidly growing population due to the massive influx of foreign labor, where expatriates constitute about 87.3% of the total population [11]. This diversity creates a number of unique healthcare challenges including the importation and spread of communicable diseases. The Ministry of Public Health (MOPH) in Qatar, in particular the Health protection & Communicable Disease Control (HP & CDC) section, has implemented a national surveillance program for influenza and other ILIs associated viruses to identify and monitor the circulating viruses in the country. In this present study, we conducted a retrospective analysis utilizing the existing data from the influenza surveillance system in order to explore the etiology and the frequency of influenza like illness among adults in Qatar.

## Methodology

### Case definition and specimen collection

Specimens were collected from patients with an age of 15 years or above and presented with ILI attending Hamad General Hospital clinics, sentinel sites, and all primary healthcare centers in Doha between January 2012 to December 2017. A standardized ILI case definition according to the WHO was strictly followed, which defines ILI as fever ≥38°C and cough or sore throat with onset within the last 10 days [12]. For viral detection, specimens from throat swabs (oropharyngeal), nasal or nasopharyngeal swabs, and nasal aspirate were collected. In addition, sputum samples were collected for follow-up analysis from patients who were identified with confirmed Middle East Respiratory Syndrome virus (MERS CoV) infection. All specimens were collected in viral transport medium, stored at 4° C, and sent to the virology laboratory of Hamad Medical Corporation (HMC) for testing.

### Laboratory testing

Tests for more than 20 respiratory pathogens are routinely performed at HMC virology lab. Following the physician’s request, specimens are either tested for influenza A/B/H1N1 only using the Xpert Flu assay (Cepheid, U.S.), or against a panel of respiratory viruses using Respiratory Pathogens 21 kit (Fast-Track Diagnostics, Luxemburg). This kit enables detection of 20 viruses ‎and one bacteria that cause upper respiratory tract infections with an overall sensitivity and specificity of 95% ‎and 99% respectively. In addition, FTD MERS-CoV kit (Fast-Track Diagnostics, Luxemburg) was used to screen for MERS-CoV. DNA/RNA was extracted on EziOne and amplified on ABI7500, which is a CE-In vitro diagnostic (IVD) approved system.

### Statistical analysis

Data, which included epidemiological and laboratory diagnostic test results, were retrieved from SARI surveillance database of the Ministry of Public Health in Qatar. Initially, we checked and cleaned the datasheets from errors and duplicates before conducting any analysis. To estimate accurately the rates of infections, subjects who had similar test results within 14 days were considered as duplicates. We conducted a descriptive statistical analysis to analyze the demographic and clinical characteristics of the study subjects; and to identify the frequency, patterns, and seasonality of all viruses. The rate of infection of each virus was calculated as the proportion of positive specimens for that particular virus out of the total tested samples per month or year. All samples (n = 43,597) were tested for influenza viruses, while 37,929 samples were subjected for the detection of influenza and other respiratory pathogens as described above. Accordingly, the rates of influenza infections were calculated out of 43,597, while infection rates of other respiratory pathogens were calculated out of 37,929. To determine the relation significance between categorical variables, Pearson chi-square or Fisher Exact test were used. Results were considered statistically significant at *p-value* < 0.05.

### Ethics statement

The study included analysis of data obtained from ILI national surveillance system without patients’ identification information. The study was approved by Qatar University, MOPH as well as HMC-MRC: approval # 16335/16.

## Results

### Demographics

A total of 43,597 specimens were collected at different surveillance sites from patients with ILIs, between January 2012 and December 2017. The age of patients enrolled in this study was 15 years or above (mean: 43 ± 17.9, median: 38). The majority aged above 50 years, or between 15 and 30 years (30.1% and 30.2% respectively). Males and females represented 36.6% (15,990) and 25.5% (11,117) of the study population, respectively. However, gender data was not available for 37.9% (16,544) of the samples, particularly between 2012 and 2015. Table 1 summarizes the demographics of the enrolled subjects.

**Table 1:** Demographic characteristics of the study population.

Category		2012 No. (%)	2013 No. (%)	2014 No. (%)	2015 No. (%)	2016 No. (%)	2017 No. (%)	Total No. (%)
**Gender**								
	Male	0	0	0	4527 (44.7)	5461 (61.3)	6002 (59.0)	15990 (36.6)
	Female	0	0	0	3491 (34.5)	3450 (38.7)	4176 (41.3)	11117 (25.5)
	Missing data	1817 (100)	4207 (100)	8357 (100)	2105 (20.8)	3 (0.04)	1 (0.01)	16544 (37.9)
	**Total**	1817 (100)	4207 (100)	8357 (100)	10123 (100)	8914 (100)	10179 (100)	43597 (100)
**Age groups**								
	15–30	534 (29.4)	1294 (30.8)	2669 (31.9)	2938 (29.0)	2832 (31.8)	2886 (28.4)	13153 (30.2)
	31–40	393 (21.6)	987 (23.5)	1908 (22.8)	2612 (25.8)	1990 (22.3)	2589 (25.4)	10479 (24.0)
	41–50	249 (13.7)	597 (14.2)	1275 (15.3)	1627 (16.1)	1172 (13.2)	1552 (15.3)	6472 (14.9)
	>50	641 (35.3)	1329 (31.6)	2505 (30.0)	2946 (29.1)	2920 (32.8)	3152 (31.0)	13493 (31.0)
	**Total**	1817 (100)	4207 (100)	8357 (100)	10123 (100)	8914 (100)	10179 (100)	43597 (100)

### Circulating viruses

Out of 43,597 samples tested throughout the whole six years, 20,278 (46.5%) were positive for at least one respiratory pathogen. Of the positive samples, influenza virus was the most frequently detected virus, reported in 9863 (22.6%) of all ILI patients, followed by HRVs and non-MERS HCoV (9.7% and 5% respectively). Among those who tested positive for influenza virus, 7602 (77.1%) were typed as influenza A and 22.9% (2261) were typed as influenza B virus. Within type A positive samples, 3774 (49.6%) were A/H1N1 and 516 (6.8%) were A/H3N2 subtype, while the rest were not tested for any subtype (Table 2). Other respiratory viruses were also reported at lower frequencies, including: adenoviruses (2.8%), HPIVs and RSV (2.6% each), HMPV (2.5%), enteroviruses (0.9%), HboV (0.7%), and parechovirus (0.1%). Additionally, M. pneumonia infections were found in 1128 (3%) of the cases.

**Table 2:** Distribution of influenza viruses by year of detection.

**Pathogen**		**Surveillance year**						
		**Total**	**2012**	**2013**	**2014**	**2015**	**2016**	**2017**
		n = 43579	n = 1817	n = 4207	n = 8357	n = 10123	n = 8914	n = 10179
		**Positive cases**						
		**No. (%)**	**No. (%)**	**No. (%)**	**No. (%)**	**No. (%)**	**No. (%)**	**No. (%)**
**Influenza A**		7602 (17.4)	260 (20.1)	614 (19.7)	1510 (22.3)	2156 (25.2)	1221 (21.2)	1841 (23.2)
	*A/H1N1*	3774 (8.7)	177 (9.7)	114 (2.7)	836 (10.0)	1576 (15.6)	258 (2.9)	813 (8.0)
	*A/H3*	516 (1.2)	§	§	§	§	§	516 (5.1)
**Influenza B**		2261 (5.2)	106 (5.8)	213 (5.1)	355 (4.2)	394 (3.9)	670(7.5)	523.1)

§ Samples were not tested for A/H3

**Fig 1:**
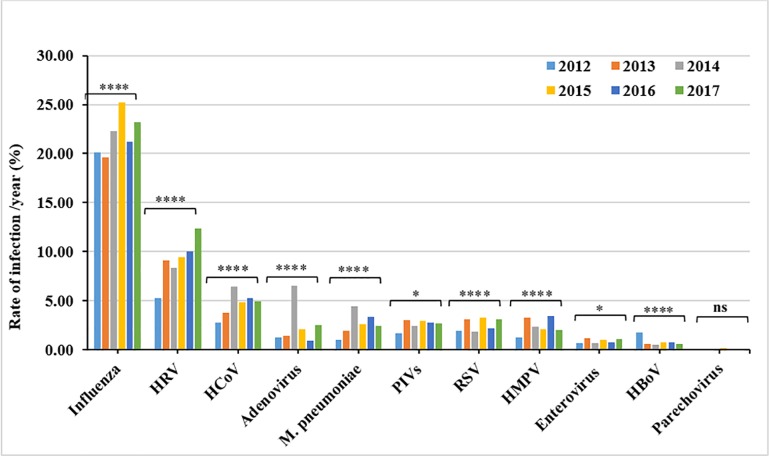
Annual rate of respiratory infections detected from 2012–2017. The rate of infection per year was calculated as the number of positive cases of each specific virus per the total number of tested samples (n = 43,597 tested for influenza viruses, n = 37929 tested for other viruses). The difference in the infection rate per year was calculated using Pearson Chi2. P-value less than 0.05 is flagged with one star (*). P-value less than 0.01 is flagged with two stars (**). P-value less than 0.001 is flagged with three stars (***). P-value less than 0.0001 is flagged with four stars (****). Ns: not significant.

Patterns of respiratory viral infections were similar throughout the six-year study period (2012–2017). While influenza viruses were the dominant cause of ILIs in all six years with rates ranging between 19.7%-25.2% of all the tested samples, HRVs and HCoVs were the second and third most frequently detected viruses, with a positivity rate ranging between 5.3%- 12.4% and 2.9%- 6.4%, respectively. Adenoviruses represented a considerable number of detections in 2014, accounting for 6.5% (Fig 1, Table 3). Notably, the highest rate of influenza viruses infections were reported in 2015, detected in 25.2% of the samples. On the other hand, M. pneumonia, adenoviruses, and HCoVs were more frequently detected in 2014 (4.4%, 6.5%, and 6.5% respectively). HRVs infections had a significant increase in 2017 compared to former years, accounting for 12.4% of tested samples.

**Table 3:** Distribution of different respiratory pathogens by year of detection.

Pathogen		Surveillance year						
		Total	2012	2013	2014	2015	2016	2017
		n = 37929	n = 1817	n = 4207	n = 8357	n = 7983	n = 7100	n = 8465
		Positive cases						
		No. (%)	No. (%)	No. (%)	No. (%)	No. (%)	No. (%)	No. (%)
**Adenoviruses**		1078 (2.8)	23 (1.3)	62 (1.5)	545 (6.5)	171 (2.1)	65 (0.9)	212 (2.5)
**HMPV**		941 (2.5)	23 (1.3)	137 (3.3)	196 (2.3)	167 (2.1)	247 (3.5)	171 (2.0)
**HRVs**		3697 (9.7)	96 (5.3)	382 (9.1)	702 (8.4)	757 (9.5)	710 (10.0)	1050 (12.4)
**Bocavirus**		268 (0.7)	32 (1.8)	27 (0.6)	45 (0.5)	59 (0.7)	55 (0.8)	50 (0.6)
**HCoV**		1902 (5.0)	53 (2.9)	158 (3.8)	536 (6.4)	377 (4.7)	369 (5.2)	409 (4.8)
	*229E CoV*	538 (1.4)	11 (0.6)	47 (1.1)	148 (1.8)	106 (1.3)	126 (1.8)	100 (1.2)
	*NL63 CoV*	244 (0.6)	6 (0.3)	23 (0.5)	71 (0.8)	36 (0.5)	44 (0.6)	64 (0.8)
	*HKU1 CoV*	391 (1.0)	10 (0.6)	30 (0.7)	124 (1.5)	87 (1.1)	64 (0.9)	76 (0.9)
	*OC43 CoV*	705 (1.9)	24 (1.3)	52 (1.2)	191 (2.3)	143 (1.8)	132 (1.9)	163 (1.9)
	*MERS-CoV*	24 (0.06)	2 (0.1)	6 (0.1)	2 (0.02)	5 (0.06)	3 (0.04)	6 (0.07)
**HPIVs**		1023 (2.3)	31 (1.7)	128 (3.0)	203 (2.4)	236 (3.0)	194 (2.7)	231 (2.7)
	*HPIV-1*	236 (0.5)	1 (0.1)	33 (0.8)	35 (0.4)	93 (1.2)	20 (0.3)	54 (0.6)
	*HPIV-2*	127 (0.3)	8 (0.4)	26 (0.6)	20 (0.2)	27 (0.3)	20 (0.3)	26 (0.3)
	*HPIV-3*	468 (1.1)	19 (1.0)	50 (1.2)	106 (1.3)	74 (0.9)	109 (1.5)	110 (1.3)
	*HPIV-4*	192 (0.4)	3 (0.2)	19 (0.5)	42 (0.5)	42 (0.5)	45 (0.6)	41 (0.5)
**RSV**		1002 (2.6)	35 (1.9)	130 (3.1)	158 (1.9)	259 (3.2)	154 (2.2)	266 (3.1)
**Enteroviruses**		348 (0.9)	12 (0.7)	51 (1.2)	56 (0.7)	82 (1.0)	57 (0.8)	90 (1.1)
**Parechovirus**		44 (0.1)	1 (0.1)	4 (0.1)	10 (0.1)	16 (0.2)	7 (0.1)	6 (0.1)
**M. pneumoniae**		1128 (3.0)	19 (1.0)	82 (1.9)	371 (4.4)	207 (2.6)	240 (3.4)	209 (2.5)

### Patterns of seasonality

Qatar is characterized by subtropical dry, hot desert climate with low annual rainfall, very high temperatures in summer and a big difference between maximum and minimum temperatures. On the other hand, winter is cooler with occasional rainfalls. The weather during spring and autumn is usually warm during the days and cooler at nights. Importantly, there are occasional drops in the temperature during cool months with heavy fog conditions. This could affect the respiratory mucosa and increase individuals’ susceptibility to infections. Also, it is known that cold temperatures facilitate the survival and spread of viruses more easily [13]. Another feature of cold months is that schools are open at that time, which also contribute to infections spread. Accordingly, we investigated the patterns of seasonality of all the tested viruses to understand the temporal circulation of respiratory pathogens.

**Fig 2:**
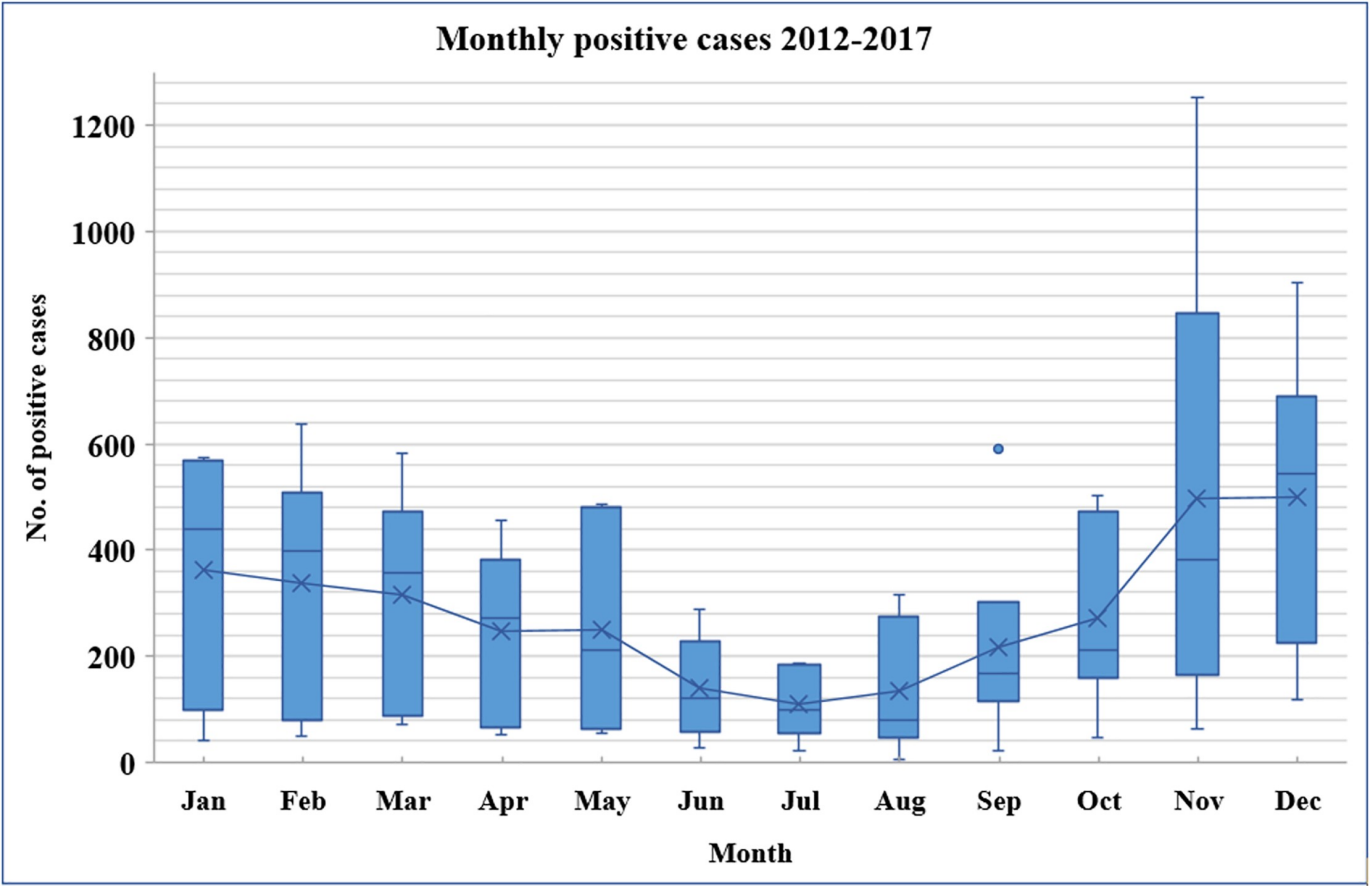
Monthly distribution of positive cases for at least one respiratory pathogen (2012–2017). The box plot shows the median mean differences. X: mean. Outer dots: outliers. Bars: standard deviation. The horizontal lines within boxes show the median.

In general, our data indicated that influenza and other respiratory pathogens exhibit strong seasonal peaks. Both, the number of reported ILI cases and the rate of positive results increased significantly during cooler months (Fig 2). Over the six-year study period, the highest number of received samples was recorded during the winter between December and February (31.3%), followed by fall months between September and November (29%). Samples received in spring and summer months represented 25.1% and 14.6%, respectively. Similarly, the overall rate of positive cases for at least one pathogen was significantly greater in winter (7205 cases; 16.5%), followed by fall (5920 cases; 13.6%), spring (4888 cases; 11.2%), then summer (2295 cases; 5%) positive cases (p <0.000).

**Fig 3:**
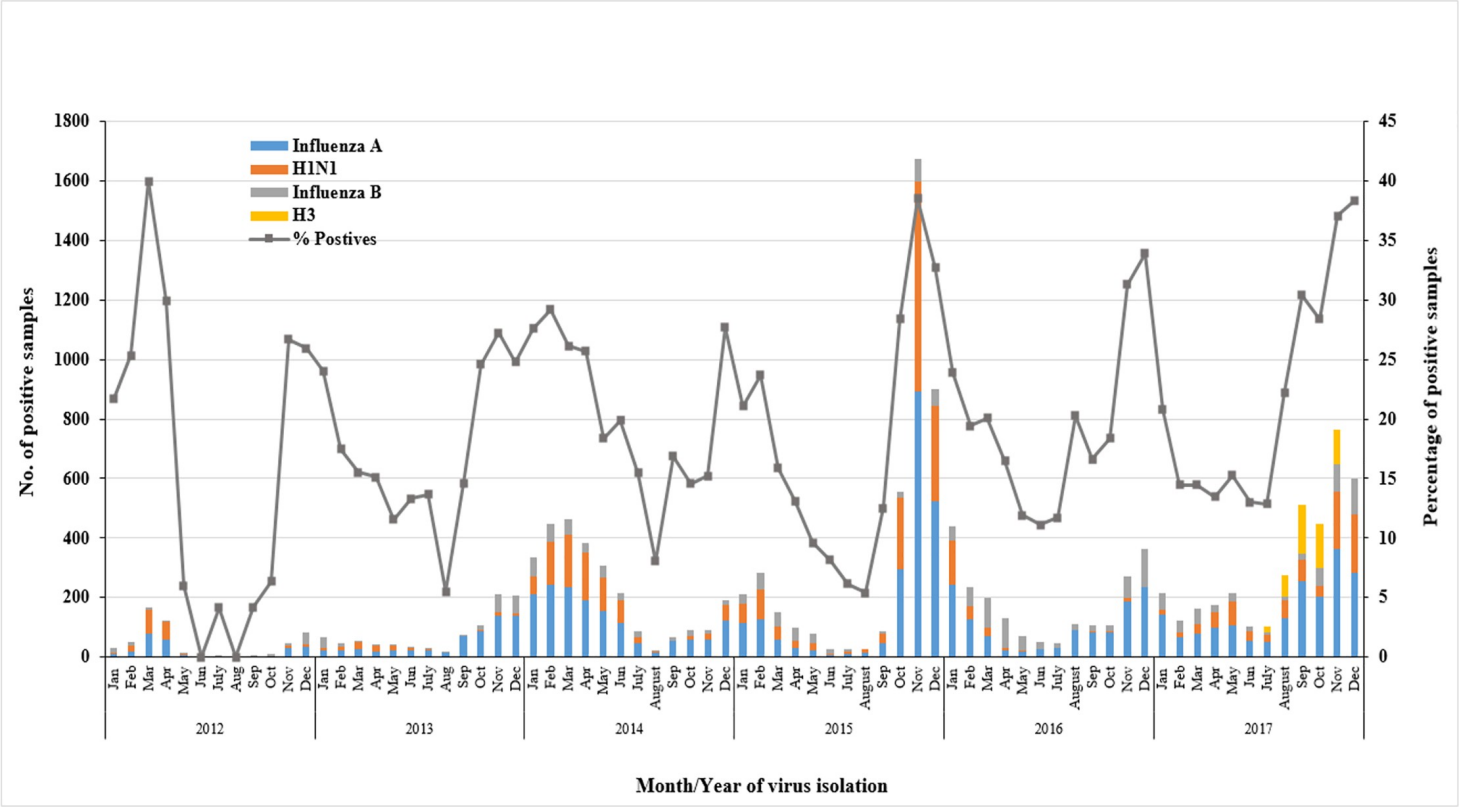
Seasonality of influenza viruses. The number of positive samples for each influenza type/subtype was calculated per month, from 2012 to 2017. The percentage of positive cases was calculated out of the total tested samples for influenza viruses (n = 43,597).

Consistently, influenza viruses were circulating throughout the year; however, data from the entire six years period clearly identified major peaks during cold months. The highest rates of influenza infections were seen between November and February during 2013 to 2017, with a detection rate up to 38.5% of all tested specimens. Interestingly, a unique pattern was observed in 2012, where the highest peaks of influenza infections was recorded during March and April (40% and 30% respectively), which declined afterwards to less than 5% starting from June to September. Subsequently, dramatic increase of influenza cases was then recorded in October and November, reaching 26.7% of tested samples. Fig 3 illustrates the seasonality of influenza viruses during all the surveillance years.

**Fig 4:**
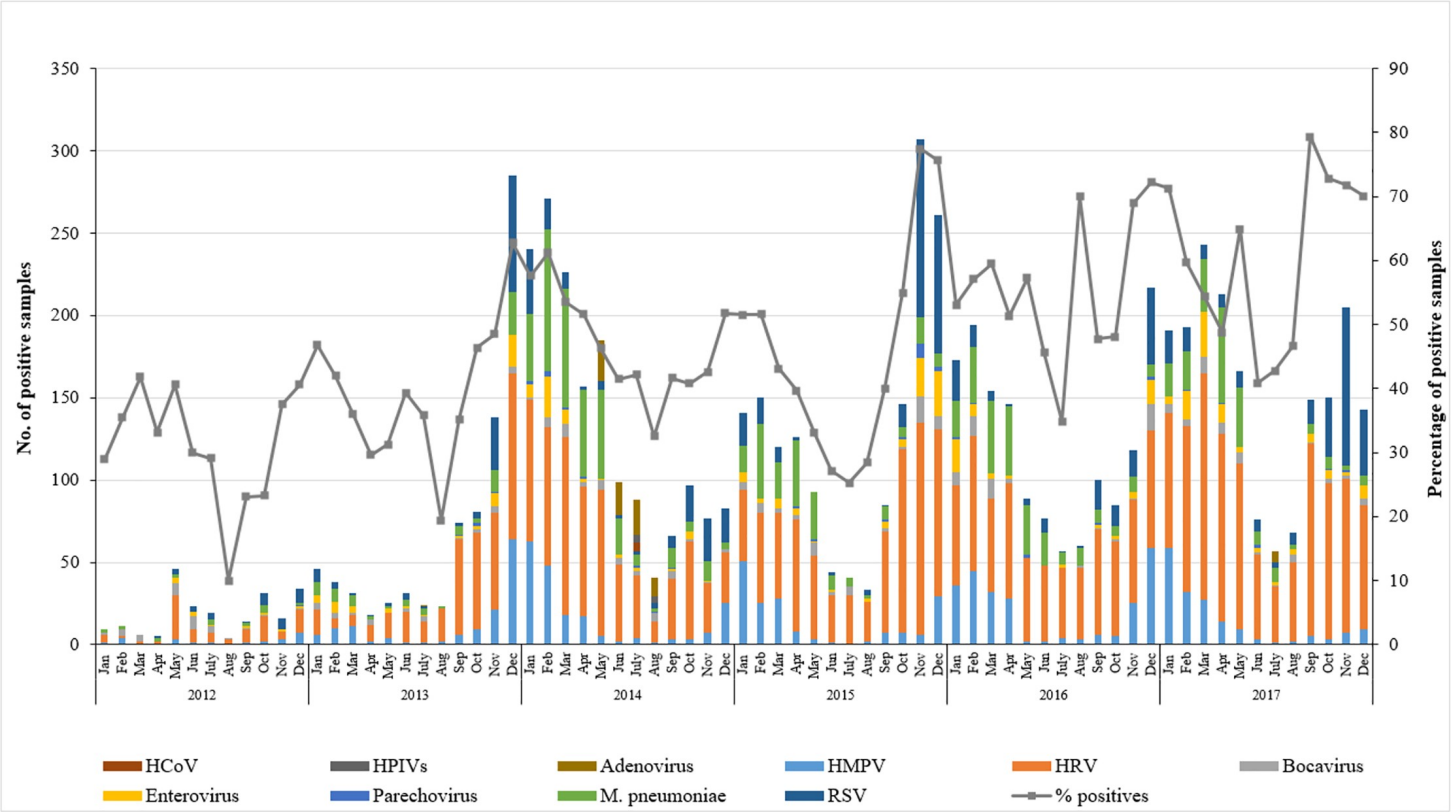
Seasonality of all ILIs causing pathogens. The number of positive samples for each pathogen was calculated per month from 2012 to 2017. The percentage of positive cases was calculated out of the total tested samples (n = 37,929).

Although the overall rate of respiratory illnesses was considerably higher in the winter, the seasonality of different respiratory pathogens was variable as illustrated in Fig 4. HRVs ranked as second leading cause of ILIs in adults. This virus tends to circulate during fall months, specifically between September and November, with a detection rate reaching 17%. Still, a high frequency of this virus was also observed in warmer months between March and May, representing 10%- 20% of tested samples. This coincided with low influenza rates around the same time of the year. Conversely, other viral agents, particularly RSV and HMPV, were predominating along with influenza viruses during the winter season and demonstrated a consistent seasonality. RSV had a unimodal peak during November or December each year, with a maximum detection rate of 9.7% recorded in 2017. On the other hand, HMPV was circulating more during January and March with a detection frequency reaching to 7.9%. M. pneumonia infection accounted for 3% of ILIs infections, in which most of the cases were detected in the period between January and April.

Virus seasonality was less interpretable for adenoviruses, coronaviruses, parainfluenza viruses (1–4), since they were distributed all over the years, with no clear temporal patterns of infection fluctuations. As for enteroviruses, HboV, and parechovirus, low rate of less than 1% positive cases were reported, with no unique cyclic occurrence.

### Correlation with demographic characteristics

We first analyzed the correlation between ILIs infections and age. The frequency of infections varied markedly between different age groups. The highest rate of influenza A viruses was observed among patients aged between 31 and 40 years, with a frequency of 19.9%. Influenza B virus was more detected among 15–30 years age group, reaching to 6.1% positive cases. To the contrary, individuals aged above 50 years had significantly lower infection rates with both influenza A (13.9%) and B (4.2%) types. Nonetheless, this age group demonstrated significantly elevated infections with RSV, HMPV, HboV, and HPIV-3 (3.4%, 3.3%, 0.8%, and 1.7% respectively), compared to younger age groups. HCoVs rates correlated variably with the age. Both 229E and HKU1 CoVs were more detected among 31 to 40 years age group, with a frequency of 1.7% and 1.2% respectively. Conversely, NL63 CoV was more commonly detected in younger individuals (15–30 age group), accounting for 0.9% infection rate. MERS CoV showed similar prevalence in both, individuals aged between 15 to 40 years and those above 50 years old (0.1%). On the other hand, OC43 CoV infection was comparable among different age groups with no significant differences (1.7–2%). Infection with HRVs, M. pneumoniae and adenoviruses inversely correlated with age, with the highest rate detected among 15–30 years age group (10.9%, 4.7%, 3.2% and 1.1%, respectively). A significant difference was also observed in the rates if infection with enteroviruses. The highest infection rate was detected in age groups below the age of 40 years (1.1% each), whereas older age groups (>40 years) recorded lower rates reaching to 0.6%. Low number of positive cases for parechovirus prohibits any correlation analysis with age (Table 4).

**Table 4:** Distribution of influenza and other respiratory pathogens by age group.

Pathogen		Total	Positive cases per age group (years)				
			15–30 No. (%)	31–40 No. (%)	41–50 No. (%)	> 50 No. (%)	*p* value*
**Influenza A**		7602	2468 (18.8)	2084 (19.9)	1180 (18.2)	1870 (13.9)	0.000
	*A/H1N1*	3774	1157 (8.8)	1169 (11.2)	649 (10)	799 (5.9)	0.000
	*A/H3*	516	164 (1.3)	139 (1.3)	77 (1.2)	136 (1)	0.120
**Influenza B**		2261	796 (6.1)	572 (5.5)	332 (5.1)	561 (4.2)	0.000
**Adenoviruses**		1078	347 (3.2)	256 (2.9)	154 (2.7)	321 (2.6)	0.034
**HMPV**		941	232 (2.1)	153 (1.7)	140 (2.5)	416 (3.3)	0.000
**HRVs**		3697	1193 (10.9)	843 (9.6)	517 (9.1)	1144 (9.1)	0.000
**HboV**		268	83 (0.8)	55 (0.6)	26 (0.5)	104 (0.8)	0.030
**HCoVs**		1902	504 (4.6)	457 (5.2)	316 (5.6)	625 (5.0)	0.049
	*229E CoV*	538	128 (1.2)	148 (1.7)	92 (1.6)	170 (1.4)	0.011
	*NL63 CoV*	244	98 (0.9)	49 (0.6)	45 (0.8)	52 (0.4)	0.000
	*HKU1 CoV*	391	87 (0.8)	109 (1.2)	57 (1.0)	138 (1.1)	0.016
	*OC43 CoV*	705	184 (1.7)	150 (1.7)	118 (2.1)	253 (2.0)	0.110
	*MERS-CoV*	24	7 (0.1)	1 (0.01)	4 (0.07)	12 (0.1)	0.119
**HPIVs**		1023	264 (2.4)	226 (2.6)	145 (2.5)	388 (3.1)	0.009
	*HPIV-1*	236	70 (0.6)	56 (0.6)	31 (0.5)	79 (0.6)	0.883
	*HPIV-2*	127	42 (0.4)	34 (0.4)	16 (0.3)	35 (0.3)	0.361
	*HPIV-3*	468	101 (0.9)	84 (1.0)	72 (1.3)	211 (1.7)	0.000
	*HPIV-4*	192	51 (0.5)	52 (0.6)	26 (0.5)	63 (0.5)	0.592
**RSV**		1002	244 (2.2)	186 (2.1)	145 (2.5)	427 (3.4)	0.000
**Enteroviruses**		348	124 (1.1)	94 (1.1)	53 (0.9)	77 (0.6)	0.000
**Parechovirus**		44	15 (0.1)	6 (0.1)	9 (0.2)	14 (0.1)	0.385
**M. pneumoniae**		1128	518 (4.7)	302 (3.4)	140 (2.5)	168 (1.3)	0.000

* Pearson Chi2 p-value

**Fig 5:**
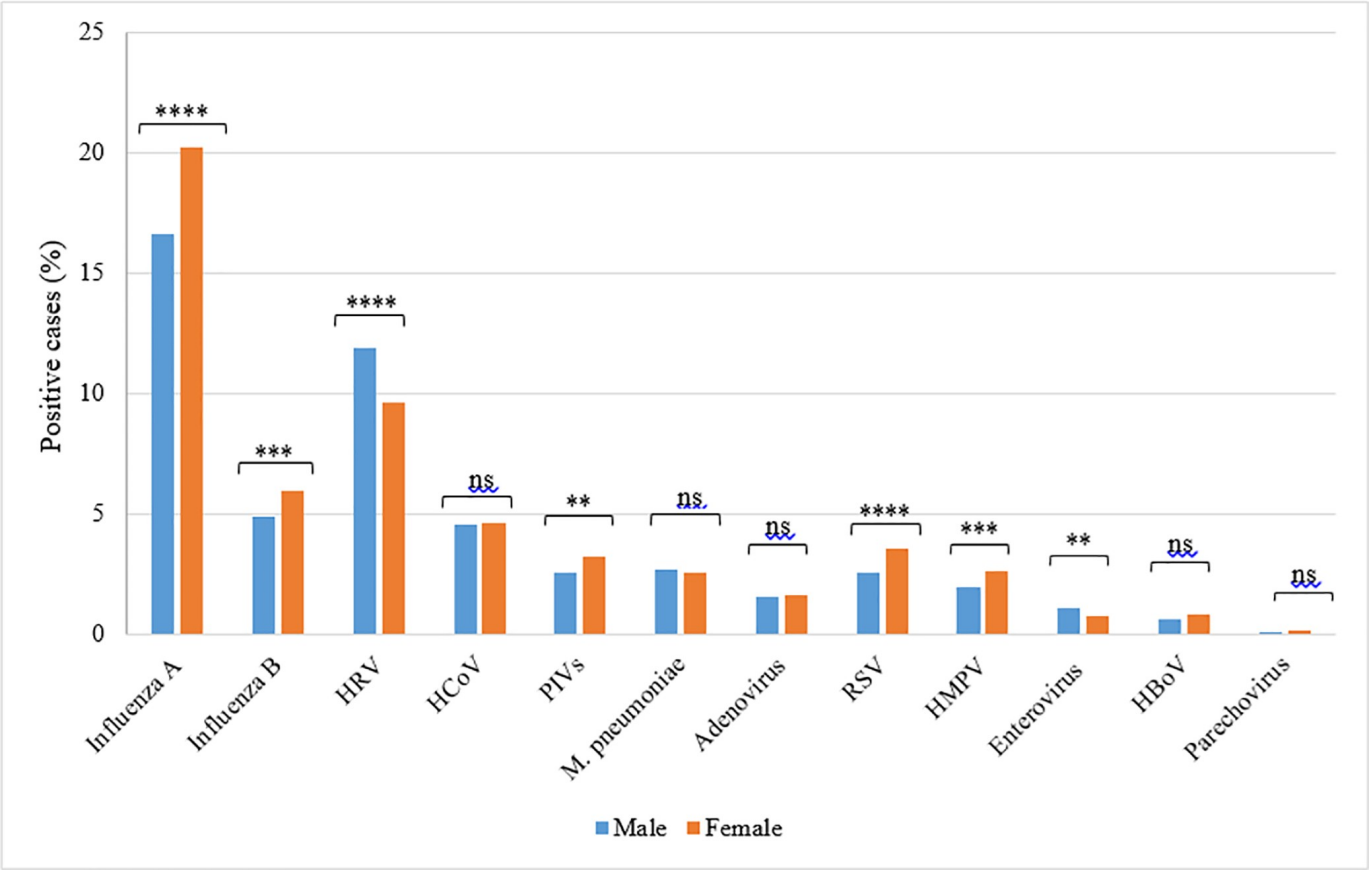
Rate of ILI infections among males and females. The difference in the infection rate between the two groups was calculated using Pearson Chi2 and the Fisher Exact test. P-value less than 0.05 is flagged with one star (*). P-value less than 0.01 is flagged with two stars (**). P-value less than 0.001 is flagged with three stars (***). P-value less than 0.0001 is flagged with four stars (****). Ns: not significant.

The association between gender and different infections was then explored for the available data. Males had a strong statistical correlation with elevated HRVs (11.9%) and enteroviruses (1.1%), compared to females (9.5 and 0.7%). On the other hand, females had significantly higher rates of influenza A, influenza B, RSV, HPIVs and HMPV infections (20.6%, 6%, 3.6%, 3.2% and 2.7% respectively) in comparison to males (16.6%, 4.9%, 2.6%, 2.6%, and 2% respectively). On the contrary, rates of non-MERS HCoV, M. pneumonia, adenoviruses, HboV, and parechovirus infections were comparable between the two groups with no significant correlation (Fig 5). Yet, MERS CoV was significantly higher among males (0.2%), compared to females (0.01%). In other words, out of 24 reported MERS-CoV, 23 (95.8%) were males, only one case (4.2%) was a female.

## Discussion

Qatar is a small country on the eastern coast of the Arabian Peninsula and it is characterized by dry, subtropical desert climate with minimal annual rainfall and extremely hot and humid summers. The weather in Qatar can be broadly grouped into two seasons: hot season between May and October, and cool season between December and February. Additionally, Qatar is a multinational country, where >85% of its population are expats arriving from several countries in MENA region and South East Asia. Furthermore, Qatar is located within the hot zone of MERS-virus circulation. MERS-CoV shedding was evidenced in 59% of dromedary camels at the central slaughterhouse in Qatar, in which five different MERS strains were co-circulating [14]. The high proportion of MERS shedding and the circulation of multiple variants emphasizes on the potential human health risk in a diverse and dynamic environment like Qatar. These are all factors that make it essential to study the epidemiology of communicable diseases, especially when considering the country’s preparation for the Football World Cup 2022. Here we report on the epidemiological and demographic characteristics of ILIs among adult patients in Qatar during the period between 2012–2017.

Our study demonstrated that respiratory viruses are commonly detected in patients presented with ILIs. We found that 46.5% of the 43,578 tested specimens are positive for at least one respiratory pathogen. This finding is consistent with previous surveillance based studies on patients from different age groups reported from multiple countries. For instance, 43% positivity rate was reported from 2,031 ILI cases in Philippines (ASIA) [15], 41.7% from a total of 580 cases in Italy (EUROPE) [16], and 42.6% out of 6,835 cases in Peru (SOUTH AMERICA) [17]. Relatively lower rates were reported in Colombia (36% in 2039 individuals) [9] and in China (34.6% of 5805 subjects) [18]. Conversely, few other reports showed higher frequencies reaching to 61% in Cameron [3], 61.8% in Brazil [10], and 75.1% in Madagascar [8]. This variation can be attributed to the differences in the size and demographic characteristics of the studied populations, length of the study, the diagnostic techniques used for virus detection, and environmental factors.

Our data support previous studies highlighting influenza virus as a global contributor to respiratory illnesses burden. We found that influenza virus was predominating among ILI cases over the six-year period, accounting for more than 22% of all tested samples. This is similar to other reports in which influenza frequency ranged from 14.8% to 30.9% [8, 10, 15, 16, 18–20]. Moreover, among typed influenza positive samples, the majority were type A, reaching 77.1%. This number matches what has been recently reported in a meta-analysis study in the Middle East and North Africa region, where among 70,532 evaluated influenza cases, influenza A and B accounted for a median proportions of 76.5% and 23.5% of cases [4].

Other respiratory pathogens co-circulated with influenza viruses but at lower rates, including HRVs (9.7%) which is the second most frequent cause of ILI, HCoVs (5%), adenoviruses (2.8%), M. pneumonia (3%), HPIVs (2.3%), RSV (2.6%), HMPV (2.5%), enteroviruses (0.9%), HboV (0.7%), and parechovirus (0.1%). These findings are consistent with similar epidemiological studies conducted on adults in other countries. HRVs were also the second leading cause of ILIs in a three-year Chinese study between 2005–2007, where the virus was detected in 6.5% of patients above 14 years of age (total number = 5808 cases). All other remaining viruses were detected with a frequency less than 5% [18]. However, higher percentages of HRVs and RSV were reported from studies where younger participants were included [3, 15]. Although it is known that parechovirus may contribute to ILIs, only limited reports investigated their epidemiological characteristics among ILI patients [21]. In the current study, parechovirus infections were the lowest amongst other viruses, with only 44 positive cases reported in all the six surveillance years. In fact, low rate of parechovirus infections is typically observed in adults compared to children, where these infections are more frequent [22]. Earlier seroprevalence studies showed that over 90% of children are exposed to parechovirus before the age of two years [23]. It is therefore possible that the low rate of parechovirus-associated ILIs in adults is attributed to the presence of sterilizing immunity that confers protection and limits the infection severity, and thus, decreases the number of hospital visits among adult patients.

Analysis of the viruses’ subtypes revealed that parainfluenza type 3 was the most common detected type, representing 45.7% of all PIV positive cases, which in accordance with previous studies [24, 25]. Among those positive for CoVs, type OC43 was the most prevalent type (36.5%), similar to reports from Thailand [26], Cameron [3], Hong Kong [27] and others [28].

We observed respiratory viral activity throughout the study period with defined peaks for multiple viruses, including influenza virus, which had higher activity between November and December. This is similar to what have been reported in other countries with similar climate around the Arabian Peninsula [4]. In addition, our data identified another major peak of influenza activity between January and March of 2012, which is interestingly similar to what have been reported in Northern Hemisphere countries around the same time [4]. Nonetheless, in this current study, influenza A subtyping was not routinely performed between 2012–2016. Hence, identifying the seasonality and circulation of each subtype was not possible.

Similar to previous reports, higher HRVs circulation was recorded in periods of low influenza activity [3, 8, 29]. This indicates that while influenza predominates in the winter, HRVs are a responsible of large ratio of ARIs during other seasons [30]. In contrast to many studies where HRVs seasonality was not clear [3, 8, 18], our data shows that infections with HRVs increase during fall between September and November, and in spring between March and May. Moreover, we found that RSV and HMPV overlapped the peak of influenza, as previously described [31]. No clear temporal patterns were observed with CoVs infections, as well as adenoviruses, HPIV, enteroviruses, and parechovirus, and this is probably due to their low infection rates. It is worth noting that the analyzed samples were collected from various primary, secondary, and tertiary healthcare facilities around Qatar, and ‎hence, we believe that there is no impact of site selection on the prevalence of the analyzed pathogens. ‎

Studying the prevalence and spread of infections within different age groups in Qatar, and the GCC countries in general, is extremely important, due to the uniqueness of this region’s population. Qatar population is mainly composed of individuals between the age of 15 and 60 years, due to the importation of massive labor force from this age group. The current median age of Qatar residents is approximately 33.22 years [32], while the average age is about 41.7 years [33]. Importantly, many of expatriates work in groups at construction sites and live in labor accommodations, facilitating the continuous transmission of infections within this age group specifically. In our study, influenza A viruses were more detected among patients aged between 31 and 40 years, while younger adults (15–30 years) had higher rates of influenza B virus. On the other hand, individuals aged above 50 years had significantly lower infection rates of both influenza types. It is well recognized that elderly are more susceptible to serious complications of influenza, which could require medical intervention or even hospitalization. However, this age group was the least affected in this study. In fact, multiple other studies from India, Canada, Japan and UK showed similar results, in which young adults presented higher rates of pandemic H1N1 infections compared to older age groups [34–37]. Moreover, in a study from the US that estimated the relative risk (RR) for influenza hospitalizations (between 2009 and 2014) in different age groups indicated that individuals <50 years had the smallest RR, while adults aged between 18 and 49 had the highest RR [38]. One of the explanations is that young individuals have higher transmission rates compared to elderly [38]. In addition, age-stratified sero-epidemiological studies suggests that elderly are more likely to have a protective neutralizing response to the virus, which probably contributes the lower rates of infection observed in some regions [39].

In contrast to influenza infections, the correlation between the frequency of other viral infections and age showed that younger (15–30 years) and the elderly adults (>50 years) are more frequently infected. Confirming others observations [18, 40], we found significantly elevated rates of RSV (3.4%), HMPV (3.3%), and HPIV-3 (1.7%) among patients above 50 years old. On the other hand, HRVs, M. pneumonia, and adenoviruses infections inversely correlated with age. These infections were mostly affecting younger adults between 15 and 30 years old (detection rates of 10.9%, 4.7%, and 3.2% respectively). Similarly, a previous study from Beijing reported the highest rates of HRVs from individuals between 14 and 25 years old [18].

Males comprise around 75% of Qatar population due to a large influx of male laborers. Expectedly, ratios of male subjects in this study were higher than females all over the study period. We explored the gender-dependent differences in infections rates. We found that males had significantly higher rates of HRVs and enteroviruses. On the hand, females showed strong correlation with high rates of influenza, RSV, HPIVs and HMPV infections. This supports previous reports highlighting the contribution of gender differences in influencing infection incidence and severity [41]. While the frequencies of non-MERS CoVs were almost similar among males and females, MERS CoV infection was significantly higher among male subjects (67%). This complies with the WHO report stating that 65% of MERS cases occurred among males [42]. This gender disparity can be attributed to the limited contact of females with camels. Although women might occasionally visit camel farms or consume camel products, but unlike men, they do not attend camels’ races, nor visit slaughterhouses [43]. Therefore, they have less chances of being exposed to the virus.

This study has some limitations. First, the surveillance data are limited to the symptomatic ILI cases. Patients who did not have the standard ILI symptoms according the WHO ILI case definition were not included. Therefore, the actual burden of respiratory infections could be underestimated. Importantly, around 53% of all samples were negative for the tested pathogens, indicating the circulation of other etiological agents that could lead to similar disease manifestations. Moreover, there were no information about the vaccination record of the enrolled patients, which prevented further analysis on vaccine coverage and efficacy in the studied population.

In summary, this is a multi-year report summarizing the epidemiological and demographic data of 43,597 ILI cases in Qatar between 2012–2017. It is the largest scale investigation of ILIs in adults in the MENA region, and thus, shall have important implications on infections’ control and prevention policies. Although the weather in Qatar is generally hot throughout the year, seasonality and patterns of respiratory infections seems similar to what have been reported in different climate regions. Although influenza viruses are the leading cause of ILI in the country, other respiratory viruses still play a major role in the etiology of acute respiratory infections in Qatar. Results from this study would help in: ‎1. Improving vaccination timing through understanding viruses’ seasonality; 2. Promoting the vaccination campaigns to prioritize high-risk groups with higher infection burden; and 3. Standardizing the diagnosis procedures by prioritizing the testing for pathogens of significant clinical and epidemiological impact.

Future studies are needed to understand the clinical significance of these pathogens in Qatar. Importantly, the organization of World Cup 2022 in a small country like Qatar (11,571 km^2^) in a cooler season (November and December) where most of the respiratory viruses peak, necessitates active surveillance program, before and at the time of event’s organization.

## Supporting information

S1 Table The total number of positive samples per month of detection. (XLSX)

S2 Table The number of positive samples for each influenza type/subtype per month of detection. (XLSX)

S3 Table The number of positive samples for each pathogen per month of detection. (XLSX)

S4 Table The percentage of positive samples for each pathogen among males and females. (XLSX)
